# The design and impact of culturally-safe community-based physical activity promotion for immigrant women: descriptive review

**DOI:** 10.1186/s12889-022-12828-3

**Published:** 2022-03-04

**Authors:** Anna R. Gagliardi, Ciara Morrison, Natalie N. Anderson

**Affiliations:** Toronto General Hospital Research Institute, University Health Network, 13EN-228 200 Elizabeth Street, Toronto, ON M5G2C4 Canada

**Keywords:** Women’s health, Immigrants, Physical activity, Health promotion, Review

## Abstract

**Background:**

Immigrant women have low rates of physical activity (PA), placing them at risk for chronic diseases. Some research suggests that strategies targeting this group must be culturally-safe and community-based. This study aimed to identify the design (i.e. characteristics) and impact of culturally-safe community-based PA promotion for immigrant women.

**Methods:**

We conducted a descriptive review by searching MEDLINE, EMBASE, SPORTDiscus, CINAHL, SCOPUS, Cochrane Library and Joanna Briggs Institute Database of Systematic Reviews from inception to June 9, 2021 for English language studies that assessed community-based PA promotion strategies targeting adult immigrants and involved at least 50% women. We compiled findings in a preliminary context-mechanisms-outcomes conceptual framework.

**Results:**

We included 13 studies published from 2004 to 2020. Three included women-only; the remainder included a median of 63% women (range 50 to 98%). Studies included immigrants from Brazil, Dominican, Columbian, Haiti, Mexico, China, Vietnam, Bangladesh, India, Pakistan, Somalia, Sudan and Turkey. All but one study (89%) significantly improved one or more outcomes: PA knowledge, PA participation and anthropometric measures (e.g. weight, BMI, blood pressure). Most (89%) strategies were multi-faceted: in-person group educational sessions reinforced by take-home educational material and/or follow-up reminder phone calls. Single strategies (e.g. mailed educational material, group educational session) also achieved beneficial outcomes. We identified 17 culturally-safe characteristics of PA promotion strategies: language of choice, based in community settings or organizations, led by lay health workers, reflected ethno-cultural linguistic expressions and PA norms, and recognized and offered solutions to barriers of PA. Findings were captured in a preliminary theory of how contextual factors (gender, intersectionality) and mechanism (culturally-safe PA promotion) may influence PA-related outcomes (PA knowledge, self-efficacy and participation; anthropometric measures, quality of life).

**Conclusions:**

This study revealed the characteristics of PA promotion strategies that significantly improved PA-related outcomes among immigrants. Given that few studies focused solely on immigrant women or reported sub-analyses, the conceptual framework generated by this study can be used in future research to more definitively establish the design and impact of culturally-safe, community-based PA promotion for immigrant women.

**Supplementary Information:**

The online version contains supplementary material available at 10.1186/s12889-022-12828-3.

## Background

Physical activity (PA) significantly reduces obesity, high cholesterol, hypertension, diabetes and depression, which are risk factors for other conditions such as osteoarthritis, cancer, dementia and heart disease [1]. PA is defined as bodily movement via skeletal muscles, a broader concept than exercise, which is planned, structured and repetitive [2]. Recent guidelines highlight that PA can be achieved in various ways (e.g. daily tasks, exercise, sport, recreation), environments (e.g. home, work, community) and contexts (e.g. leisure, household, transportation, occupation) [3]. One in four adults worldwide do not meet recommended levels of PA and have a 20 to 30% increased risk of death compared with active persons, contributing to 5 million deaths annually that could be prevented [4]. The World Health Organization Global Action Plan on Physical Activity 2018-2030 emphasized the need for research on how to raise awareness and knowledge of the multiple benefits of PA [4]. The same report underscored the importance of a rights-based approach to address disparities in PA participation caused by socioeconomic determinants.

Population-level data showed that Canadian immigrant women, both newcomers and established, had lower PA rates than immigrant men or non-immigrants [5]. The same was found to be true of immigrant women in Australia, Europe and the United States [6–8]. A systematic review of 22 studies published since 2000 identified numerous determinants of PA participation among immigrant women including individual (lack of time, health status, motivation), familial (family support) and environmental (seasonality, neighbourhood, social support) factors [9]. Other studies involving qualitative interviews revealed that gender (sacrifice self-care for household/family), culture (PA not encouraged) and socioeconomic (low-paying/multiple jobs) factors contribute to low PA rates among immigrant women [10,11]. Immigrant women are also hard-to-reach due to low help-seeking arising from mistrust of healthcare providers and poor healthcare experiences [12]. We conducted focus groups with 23 immigrant women aged 25 to 78 from 10 countries who said that physicians rushed through discussions, did not address their questions, and did not make time for lifestyle counseling, which they desired as a means of preventing illness [13].

Considerable research has evaluated interventions to promote PA to patients or the public such as structured or facilitated exercise programs, but these were not consistently effective as participation waned due to intervention burden, and more importantly, did not accommodate gender, culture or socioeconomic factors that limit PA among immigrant women [14]. Instead, interventions are needed further upstream to create awareness about the importance of PA in preventing chronic diseases and impart knowledge on how to weave PA through daily activities. Community-oriented PA outreach, referring to strategies delivered in and by community agencies or representatives of target groups, may be a promising approach as some research found that community-based health promotion was more impactful than mass media or the Internet at raising awareness among immigrant groups about dementia [15]. Outreach to immigrant women must also be “culturally safe” [16–18]. Cultural safety has been defined as effective care of a person from another culture as determined by that person, where culture includes but is not limited to: age or generation, gender, sexual orientation, occupation, socioeconomic status, ethnic origin, migrant experience, religious/spiritual beliefs or disability [19,20]. In qualitative interviews, East and South Asian immigrant women in Canada said that health promotion should take place in familiar community settings, include women only, and address sociocultural and socioeconomic barriers [21,22].

PA promotion is particularly crucial for groups at high risk of chronic diseases who experience disparities in PA participation. This includes immigrant women who face a plethora of barriers to PA [9–13]. We lack knowledge of how best to promote awareness of the importance of PA to immigrant women, but research suggests that community-based culturally-safe approaches are needed. The goal of this research was to review published research and generate insight on the design (i.e. characteristics) and impact (i.e. on PA and related outcomes) of culturally-safe community-based PA promotion strategies targeted to immigrant women. If found, exemplar strategies could be broadly emulated, or if lacking, this would justify primary research to develop, implement and evaluate culturally-safe PA promotion strategies for immigrant women.

## Methods

### Approach

We conducted a descriptive review to describe the design and impact of PA promotion strategies targeting immigrant women [23]. This type of review identifies essential intervention characteristics by transforming existing empirical evidence into a conceptual framework or by mapping constructs studied to an existing framework. Thus, this approach was more suitable than a scoping review, which aims to describe the available evidence on a given topic [24], or a realist review, which builds on a proposed context-mechanism-outcomes framework, such as that generated by a theoretical review [25]. In this review, we focused on transforming empirical evidence into a conceptual framework. We did not analyze theories employed by included studies or others relevant to this topic because that was beyond the scope of the current review, and better addressed with other synthesis approaches such as a concept analysis. Instead, we compiled characteristics of PA promotion strategies associated with beneficial outcomes. As there are no reporting criteria specific to descriptive reviews, we complied with the Preferred Reporting Items for Systematic Reviews and Meta-Analyses [26]*.* We did not require research ethics board approval as data were publicly available and we did not register a protocol.

### Eligibility criteria

We employed the widely-used participants, intervention, comparisons, outcomes framework to categorize inclusion criteria. Participants included immigrant women aged 18+ originating from any country or origin who were well or with a specific disease/condition. Immigrants were defined as persons coming to live permanently in a foreign country [27]. To be comprehensive, we included studies involving immigrants in general provided they reported the number of women participants and women comprised at least half of the participants. Participants also included healthcare professionals, managers, policy-makers or others involved in health promotion. The intervention included single- or multi-faceted approaches, strategies, programs, interventions or tools that promoted PA to immigrants offered in community settings (including home or workplace) by healthcare professionals, community agencies or peer leaders. Promotion was defined as a range of strategies that enable people or communities to increase control over and to improve their health [28]. In particular, we were interested in educational (provide learning on health topics) and social (share information through interaction) strategies but remained open to other options. Comparisons referred to studies that explored views about the optimal design of PA promotion to immigrants, evaluated the use of impact of PA promotion strategies alone or in comparison with one or more other approaches, or identified barriers of PA promotion use or impact. This included studies of a quantitative, qualitative or mixed−/multiple-methods research design published in English language. Outcomes included desired or ideal design of PA promotion; enablers or barriers of PA promotion use or impact; or the impact of PA promotion including but not limited to PA awareness, knowledge, beliefs, attitudes, help-seeking, intention to participate in PA or PA participation. We excluded studies that focused on: refugees, a group distinct from immigrants by virtue of the health issues they face (e.g. trauma, infectious diseases); immigrant children or youth as they may require PA promotion strategies that differ from adults; strategies based largely on structured PA or exercise sessions rather than promotion of PA; or strategies based on policy or modifications to the build environment as our focus was on strategies directly aimed at immigrant women (e.g. educational, social). We excluded publications in the form of letters, editorials, abstracts or protocols. We did not include systematic reviews, although we perused the references of relevant reviews to identify eligible primary studies. Additional file 1 details eligibility criteria.

### Searching and screening

NA (Research Associate) and ARG (Principal Investigator with medical librarian training) developed a search strategy that captured the concepts of physical activity, immigrants and health promotion (Additional file 2) that complied with the Peer Review of Electronic Search Strategy reporting guidelines [29]. We searched MEDLINE, EMBASE, SPORTDiscus, CINAHL, SCOPUS, the Cochrane Library and Joanna Briggs Institute Database of Systematic Reviews from database inception to June 9, 2021. To pilot test the screening process, CM (Research Assistant), BR (Research Assistant), NA and ARG independently screened the first 50 titles and abstracts, then compared and discussed discrepancies to achieve a common understanding of how to apply eligibility criteria. Thereafter, CM screened remaining titles and abstracts, and discussed uncertainties with NA and ARG to resolve eligibility decisions. CM retrieved and screened full-text articles concurrent with data extraction, and consulted with NA and ARG to resolve uncertainties.

### Data extraction and analysis

CM, NA and ARG pilot-tested data extraction on two studies, and compared and discussed discrepancies to achieve a common understanding of what data to extract and how. Thereafter, CM extracted and tabulated data, and discussed uncertainties with NA and ARG, who reviewed all extracted data. We did not assess methodological quality of included studies as that is not required of descriptive reviews [23]. We extracted data on study characteristics: author, publication year, country, objective, research design and participants. We described PA promotion strategy design using the Workgroup for Intervention Development and Evaluation Research framework, which captured details about community-based orientation: content, format, delivery, timing and personnel [30]. We also described cultural safety of desired or tested strategies [19–22]. While there is no established theory or model of cultural safety, we extracted details as provided by authors on theory underlying strategy design and tailoring of the strategy to make it suitable or acceptable to immigrants or immigrant women. Findings included strategy impact, enablers or barriers, or other results (e.g. views about desirable strategy design). We used summary statistics to describe study characteristics, and reported other study details and finings using tables and text.

## Results

### Search results

**Fig. 1:**
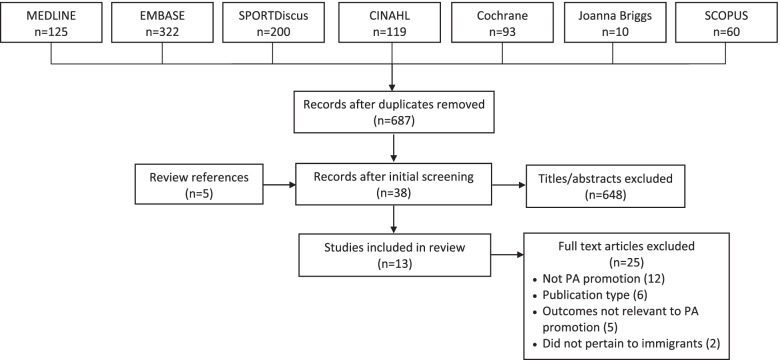
PRISMA diagram. Details number of articles retrieved from searching, eliminated through screening, and ultimately included in the review

We identified a total of 929 studies and 5 primary studies in systematic review references, from which 687 unique studies remained after removing duplicates. Screening of titles and abstracts eliminated 648 studies. Among 38 full-text articles screened, we excluded a total of 25 studies that did not focus on a PA promotion strategy (*n* = 12), meet publication type criteria (*n* = 6), report outcomes related to PA promotion (*n* = 5) or pertain to immigrants (*n* = 2). By screening the references of 3 reviews, we identified 5 unique eligible primary studies. In total, we included 13 studies (Fig. 1). Additional file 3 reports data extracted from included studies [31–43].

### Study characteristics

Studies were published from 2004 to 2020. Most were conducted in the United States (11, 84.6%), followed by 1 (7.7%) each in Germany and Norway. With respect to their objective, 4 (30.8%) studies developed a strategy to promote PA, and all employed qualitative approaches, either focus groups alone, or meetings and focus groups. Another 9 (69.2%) studies evaluated a strategy to promote PA. Of those, the research design was a randomized controlled trial in 7 studies and a before-after cohort in 2 studies. Among 13 included studies, 10 (76.9%) aimed to improve healthy eating in addition to PA. In the 4 studies that developed strategies, 1 (25.0%) study involved women only (*n* = 20). In the remaining 3 studies, the median number of participants was 25 (range 8 to 46) and the median proportion of women was 63% (range 50 to 68%). In the 9 studies that evaluated strategies, 2 (22.2%) studies involved women only (*n* = 215 in one, *n* = 198 in the second). In the remaining 7 studies, the median number of participants was 178 (range 26 to 725) and the median proportion of women was 63% (range 50 to 98%). No studies that included both women and men reported sub-analyses by sex. Among 13 included studies, 6 (46.2%) focused on immigrants from Latin countries (e.g. Brazil, Dominican, Columbian, Haiti, Mexico). Other ethno-cultural target groups included immigrants from East (China, Vietnam) and South (Bangladesh, India, Pakistan) Asia, Africa (Somalia, Sudan) and Europe (Turkey). A few (4, 30.8%) studies employed formal theory to design and/or evaluate the strategy including: Adult Learning Theory, Diffusion of Innovations Theory, Social Cognitive Theory, Social Learning Theory and the Transtheoretical Model.

### Developing strategies

Four qualitative studies explored or developed strategies to promote PA: two involved social media or Internet [34,36], and two involved community-based education sessions [41,42]. Focus groups with 25 (68% women) Asian and Hispanic immigrants in the United States revealed perceived benefits (e.g. read information at their own pace) and barriers (e.g. lack of technology or Internet, work and family obligations, privacy concerns, age-related proficiency) of social media [34]. A focus group with 8 (63% women) Turkish immigrants in Germany explored views about a bilingual web site that provided tailored guidance based on sex, age or weight [36]. Those participants appreciated that it was bilingual, advice was tailored, and it was easy to navigate, but recommended including PA options that were culturally appropriate (not specified). Focus groups with 20 Latin women in the United States assessed views of a video-taped, language-preferred (Spanish, Portuguese, Haitian Creole) didactic and interactive educational session, and their feedback (not specified) was to be used to further refine the strategy [41]. In that study, the educational sessions had been developed in consultation with women from those ethno-cultural communities and representatives of community-based organizations, and emphasized preferred language, interactive group sessions, and finding solutions to barriers of PA. Focus groups with 46 (50% women) Chinese immigrants in the United States and Canada explored preferred content (e.g. benefits of PA, solutions for common barriers, list of community resources) and format of an educational session (e.g. interactive guided discussion, audio-visual aids, videos) [42].

### Evaluating strategies

Of the 9 studies that evaluated strategies, 4 (44.4%) studies explicitly mentioned employing community-based participatory research [32,37,38,43], yet all engaged community agencies or representatives of target groups in developing and delivering strategies. All strategies were delivered in the participants’ language. One (11.1%) study evaluated printed educational material that was mailed to Latin women in the United States on 26 occasions over a one-year period [39]. Content included PA advice, local locations, log sheets, individually-tailored feedback and motivational messages based on participants’ responses to monthly questionnaires, and feedback on progress over time. The remaining 8 (88.9%) studies evaluated community-based in-person group education sessions delivered in the home (*n* = 1) [32], at work to farmworkers (*n* = 1) [35], and in community organizations such as immigrant settlement services or culturally-focused support agencies (*n* = 6 )[31,33,37,38,40,43]. Of those 8 studies, 6 (75.0%) employed trained lay health workers of the same ethno-cultural background as participants to deliver educational sessions, and 6 (75.0%) involved multifaceted strategies where educational sessions were combined most often with educational material (e.g. booklet of key messages, list of community resources) and follow-up reminder telephone calls. These studies offered a median of 6 educational sessions (range 2 to 10) over a median 4-month (range 1 to 12) period of time. In addition to recommendations and examples of PA, session content most commonly included the link between PA and disease risk and prevention, goal-setting, and solutions to barriers of PA. Additional file 4 reports the design of strategies that were evaluated in 9 studies.

### Strategy impact

Overall, 8 (88.9%) studies showed significant improvement in one or more impacts. Knowledge of PA significantly improved in 3 studies that assessed knowledge [31,33,37]. Anthropometric measures significantly improved in 3 of 5 studies that assessed measures such as weight, BMI, waist circumference, blood pressure, etc. [35,38,40]. Self-reported PA significantly improved in 5 of 7 studies [31,35,37,39,43]. PA did not improve in the single study that assessed it objectively by accelerometer [32]. There were no readily discernable differences in strategies employed by 3 studies that failed to improve PA as measured by self-report or accelerometer compared with 5 studies that improved PA: 1 provided 6 home-based lay-led educational sessions plus reminder phone calls to Hispanic, Somali and Sudanese immigrants [32], 1 provided 2 community-based lay-led educational sessions plus reminder phone calls to Chinese immigrants [33], and 1 provided 6 community-based educational sessions plus reminder phone calls to South Asian immigrants [38]. Additional file 3 reports details of the impact of strategies evaluated in 9 studies. Table 1 summarizes impacts.

**Table 1:** Summary of the impact of strategies to promote physical activity

Study Research design Strategy	Strategy Ethno-cultural group	Knowledge	Anthropometric measures	Physical activity (self-reported)	Physical activity (measured)
Jih [31] Trial Multi-faceted	Community-based education (lay health worker) Vietnamese (50% women)	✓	–	✓	–
Weiland [32] Trial Multi-faceted	Community-based education (home, lay health worker) Hispanic, Somali, Sudanese (60% women)	–	X	–	X
Jih [33] Trial Multi-faceted	Community-based education Chinese (81% women)	✓	–	X	–
Mitchell [35] Trial Single	Community-based education (workplace, lay health worker) Latinos/Latinas (72% women)	–	✓	✓	–
Islam [37] Cohort Multi-faceted	Community-based education (lay health worker) Bangladeshi (58% women)	✓	X	✓	–
Kandula [38] Trial Multi-faceted	Community-based education South Asians (East Indian, Pakistani) (63% women)	–	✓ (not all measures)	X	–
Marcus [39] Trial Single	Mailed print educational material Latinas (100% women)	–	–	✓	–
Telle-Hjellset [40] Trial Multi-faceted	Community-based education Pakistani (100% women)	–	✓ (not all measures)	–	–
Kim [43] Cohort Single	Community-based education (lay health worker) Latinos/Latinas (98% women)	–	–	✓	–

### Strategy cultural safety

Table 2 summarizes 17 culturally-safe characteristics of PA promotion strategies that appear to be important, either because they were desired by participants of studies that developed strategies, or featured in strategies that were evaluated and found impactful in most (88.9%) studies. Notably, strategies were often co-designed with input from community advisors representing the target immigrant group. Content included language of choice, linguistic appropriateness in terms of vernacular or expressions, appropriate examples of PA, discussion of solutions to overcome common barriers and lists of locally-available venues or settings of PA. Specific barriers of PA articulated by women included: little control over time due to caregiver or household obligations, little family support, lack of child care, cultural norms about self-sacrifice, dealing with inclement weather and neighbourhood safety [37,39,41,43]. Format refers to educational sessions, use of both didactic (e.g. lectures) and interactive (e.g. discussion, games) components, visual aids such as videos or pictures to combat low literacy even in participant’s first language [40] and take-home educational material. Delivery was in-person, in groups, and reinforced with individualized follow-up one-on-one or sessions or reminder phone calls. Notably, in two studies, participants mentioned barriers of Internet- or social media-based strategies [34,39], and in one study, phone calls were said to be disruptive to family time or schedules [39]. Women in particular said they preferred group activities where they could interact with other women [41]. With respect to Timing, strategies offered multiple sessions, they were spaced over weeks or months, with opportunity to re-do sessions that were missed. In one study, over 75% of participants reported that they enjoyed making promises after each session and reviewing them at the next session [35], and in another study involving 10 educational sessions, most (89 to 96%) participants reported that the number of sessions and total months involved were “just right” as opposed to “too many” or “too few [37]. Personnel referred to sessions taking place in familiar community settings or organizations, and led by lay health workers representing participant’s ethno-cultural group. Lay health workers possessed an “intimate understanding of community sociocultural background, experiences and challenges” [43], and said that “people are willing to be more honest with me than they are with doctors” and “people feel confidence in me to share things.” [37].

**Table 2:** Culturally-safe characteristics of physical activity promotion strategies

Design	Characteristics
Content	• Bilingual or choice of preferred language
	• Ethno-culturally appropriate linguistic expressions
	• Ethno-culturally appropriate examples of physical activity (options, pictures)
	• Discussion of solutions to overcome barriers
	• List of community resources where physical activity occurs
Format	• Educational sessions
	• Both didactic and interactive components
	• Visual aids such as videos and pictures
	• Take-home educational material
Delivery	• In-person
	• Group sessions
	• Reinforced by follow-up to individuals via one-on-one sessions or reminder phone calls
Timing	• Multiple educational sessions
	• Spaced over weeks or months
	• Opportunity for make-up sessions that were missed
Personnel	• Take place in familiar community settings or organizations
	• Led or co-led by lay health workers representing participant’s ethno-cultural group

Based on these findings, Table 3 depicts a conceptual framework of how contextual factors (e.g. gender, intersectionality) and cultural safety design features of PA promotion strategies (e.g. content, format, delivery, timing, personnel and engagement of community agencies or individuals representing target groups) may influence a range of impacts including PA knowledge, self-efficacy and participation, and impacts of PA such as anthropometric measures and quality of life. The components of this conceptual framework must be tested through further research, but aspects of cultural safety summarized here were fairly consistent across studies and associated with numerous beneficial outcomes.

**Table 3:** Conceptual framework of the impact of culturally-safe physical activity promotion strategies. Depicts how contextual factors and cultural safety design features of PA promotion strategies may influence a range of impacts

CONTEXT	+	MECHANISMS	➔	OUTCOMES
• Gender (roles, identities) • Intersectional factors (age, ethnicity, culture, education, literacy) • Personal, ethno-cultural, socioeconomic, family or environmental factors that facilitate or challenge physical activity		CULTURAL SAFETY Content • Preferred language • Linguistic expressions • Physical activity options • Solutions to barriers • Community resources Format • Educational sessions • Didactic and interactive elements • Visual aids • Take-home educational material Delivery • In-person • Group sessions • Follow-up: one-one-one sessions or reminder phone calls Timing • Multiple sessions • Spaced over weeks or months • Make-up sessions Personnel • Co-design/community-based participatory research • Community settings/organizations • Led by lay health workers		• Knowledge about physical activity benefits and options • Self-efficacy to participate in physical activity • Intent to participate in physical activity • Physical activity (self-reported or objectively-measured) • Anthropometrics (e.g. weight, BMI, waist circumference, blood pressure, blood glucose, cholesterol) • Quality of life

## Discussion

The goal of this study was to review published research and generate insight on the culturally-safe design and impact of community-based PA promotion strategies targeted to immigrant women. A total of 13 studies yielded 17 characteristics of culturally-safe strategies that promoted PA to immigrants from multiple countries. Most strategies were multifaceted, and in addition to in-person group educational sessions, often included take-home educational material and follow-up phone reminders. Common cultural safety features were language of choice, based in community settings or organizations, led by lay health workers, reflected ethno-cultural linguistic expressions and PA norms, and recognized and offered solutions to barriers of PA. All but 1 study (8, 89%) significantly improved PA knowledge, anthropometric measures, and/or PA participation. While few (3, 23%) studies focused solely on immigrant women, the findings are relevant to immigrant women, as they comprised a median of 63% (range 50 to 98%) of participants in studies that also included men.

Prior reviews have identified factors that influence immigrant participation in sport and physically activity [44]. In contrast, our review was undertaken to address the fact that PA rates among immigrant women are known to be low, and specifically sought to identify how to promote PA to women in community settings. These findings confirm prior research that revealed the value of community-oriented approaches for health promotion outreach to immigrants on the topic of dementia [15]. This study also expanded our understanding of what constitutes cultural safety, particularly in the context of PA promotion. In prior research involving qualitative interviews, East and South Asian immigrant women identified three aspects of health promotion that would appeal to them: take place in familiar community settings, include women only, and address sociocultural and socioeconomic barriers [21,22]. Our study identified 17 characteristics of culturally-safe strategies to promote PA to immigrants. A study involving 19 South Asian Muslim women in Canada improved self-efficacy, aerobic capacity and quality of life who participated in a 24-week mosque-based exercise intervention, emphasizing the importance of cultural safety and community access even for structured PA programs [45]. Ahmad et al. interviewed 46 Chinese and South Asian immigrant women living in Canada less than 5 years, which revealed lack of familiarity with health system-based health promotion and lack of comprehension of those health messages, and greater familiarity with informal means of obtaining health information through social networks [46]. Hence, this research addressed a gap in knowledge about how to promote PA to immigrant women by revealing concrete information about culturally-safe characteristics of impactful, community-based PA promotion strategies, knowledge synthesized in a context-mechanism-outcome conceptual framework.

Several key features of PA promotion appear to be important. Participants valued in-person group educational sessions, particularly women. While some used social media, they reserved this for communication with family and friends, and access to technology or the Internet was variable. Research on use of the Internet as a source of health information among immigrants offers mixed findings. For example, while two-thirds of American Korean women had acquired health information from the Internet, less than 40% were confident in using the Internet for this purpose [47]. A survey of 1273 American Latinos found that 44% had Internet access, and among those, use of the Internet to acquire health information varied by age, acculturation, education, self-efficacy and trust in online health information [48]. Other research revealed that immigrants, particularly women, appreciated in-person interaction with others as a source of support [49]. Thus, PA promotion is likely best offered via in-person educational settings rather than via the Internet because immigrants find this format to be appealing and supportive, and it reduces socio-economic inequities in access to health information.

A systematic review of 40 trials published from 2020 to 2015 found that lay health workers had a positive impact on self-management of chronic conditions among the general population with diabetes or cardiovascular conditions, although the role of lay health workers varied widely across studies [50]. In our study, lay health advisors were trained to lead or co-lead educational sessions, and in some cases, to conduct follow-up reminder phone calls. Because lay health workers represented participant’s ethno-cultural group, they understood socio-cultural issues and challenges, which prompted trust and confidence among participants. Another review of 26 studies published up to 2008 on the roles of lay health workers found they achieved trust by sharing personal experiences, and possessing insight on when and how to share information [51]. In addition to lay health workers, community organizations facilitated recruitment for and hosted educational sessions, but they also functioned as partners in planning and developing PA promotion strategies. This type of collaboration, sometimes referred to as community-based participatory research, is known to generate insight or products that are more likely to be relevant to target users [52,53], and it is well-established that interventions co-designed and tailored to address pre-identified barriers are more likely to be impactful [54].

While prior research established that single interventions were just as effective as multifaceted approaches [55], this study found that successful strategies reinforced learning acquired through educational meetings with take-home educational material and follow-up reminder phone calls. Notably, 3 of 9 studies in our review involved single strategies, and all achieved beneficial outcomes including increased PA participation. Hence, further research may be needed to establish the value of single versus multi-faceted PA promotion to immigrant women. Further research is also needed to assess the impact of PA promotion on outcomes such as satisfaction with and participation in educational sessions, and on PA assessed using multiple measures, which is recommended [56]. Ongoing research should also build on the preliminary theory generated here, or identify other theories useful to the study of PA promotion to immigrant women, as few studies in this review used formal theory to inform their work, further signaling the need for such theory [57].

Strengths of this research included use of rigorous synthesis methods [23], and reporting of methods and results in compliance with standards for reviews and search strategies [26,29]. By choosing a descriptive approach for this review, we fully described strategy features that support cultural safety, and captured those features in an early theory of culturally-safe PA promotion for immigrant women. Some limitations must be mentioned. As is typical for any review, the search strategy may not have identified all eligible studies. For example, we may not have identified studies targeting socio-economically disadvantaged women if they were not indexed as being about immigrants. Also, inclusion of only English language studies may have eliminated relevant studies published in other languages. Although included studies were few, we maintained a broad scope and included studies involving both women and men, yielding insight that can be widely applied to the design of PA promotion for adult immigrants, and also highlighting the paucity of research on PA promotion for immigrant women, a group particularly at-risk for low rates of PA [9–13]. Studies were largely based in the United States, so strategies may not be relevant to immigrants in other countries. While studies included participants from multiple ethno-cultural groups, primary research may be needed to develop and evaluate PA promotion strategies for other immigrant groups.

## Conclusions

We identified 17 characteristics of culturally-safe, community-based PA promotion strategies relevant to immigrant women. While studies were largely conducted in the United States, participants included immigrants from multiple countries, so the findings may be broadly relevant. Strategies largely consisted of in-person group educational sessions, often reinforced with take-home educational material and follow-up reminder phone calls. Most strategies were developed and delivered in partnership with community agencies or representatives of target groups. Strategies achieved many beneficial outcomes including increased PA knowledge, PA participation and anthropometric measures. Key features of culturally-safe PA promotion were: language of choice, based in community settings or organizations, led by lay health workers, reflected ethno-cultural linguistic expressions and PA norms, and recognized and offered solutions to barriers of PA. Findings were captured in a preliminary context-mechanisms-outcomes conceptual framework that can inform future research on PA promotion to immigrant women.

## Supplementary Information

**Additional file 1.**

**Additional file 2.**

**Additional file 3.**

**Additional file 4.**

## Data Availability

All data generated or analysed during this study are included in this published article and its supplementary information files.

## Abbreviation

PA: Physical activity
