# Supplementary material for: The design and impact of culturally-safe community-based physical activity promotion for immigrant women: descriptive review
Source: BMC Public Health. 2022 Mar 4;22:430. doi: 10.1186/s12889-022-12828-3 (PMC8895569; doi:10.1186/s12889-022-12828-3)
Supplement: Supplementary file 1 — Additional file 1. [file 12889_2022_12828_MOESM1_ESM.docx]

**Additional File 1. Eligibility Criteria**

**PURPOSE**

- The goal of this research is to review published research and generate insight on the design and impact of PA health promotion strategies targeted to immigrants, and if and how strategies considered cultural safety
- If found, exemplar strategies could be broadly emulated, or if lacking, this would justify primary research to co-produce insight on culturally safe PA health promotion strategies with immigrant women

**CONCEPTS**

**Physical Activity**

Any bodily movement produced by skeletal muscles that requires energy expenditure. Physical activity refers to all movement including during leisure time, for transport to get to and from places, or as part of a person’s work. Both moderate- and vigorous-intensity physical activity improve health. Popular ways to be active include walking, cycling, wheeling, sports, active recreation and play, and can be done at any level of skill (WHO). Physical activity differs from exercise, which is considered planned, structured and purposeful physical activity. The goal of exercise is to increase physical fitness. Eligible articles may use both terms (PA, exercise) interchangeably, so we must search for and include both.

**Health Promotion**

The process of implementing a range of social and environmental interventions that enable people and communities to increase control over and to improve their health. Health promotion can include promoting healthy behaviours, creating supportive environments and encouraging healthy public policies.

**Health Education**

One strategy for implementing health promotion programs. Health education provides learning experiences on health topics.

**Physical Activity Health Education**

Any combination of learning experiences designed with a view to facilitating voluntary actions conducive to health (e.g. counseling, discussions, individual or group meetings, telephone calls and use of written material for stimulating increased physical activity levels during leisure time or for transportation are actions relating to health education).

We are not interested in environmental interventions (e.g. building parks, walking trails, community centres, etc.), sometimes referred to as the “built environment”. We are interested in strategies that create awareness about the importance of and need for PA that may include, but are not limited to (our search may identify additional relevant approaches that we are not yet aware of):

- communication (e.g. small/large media, which includes radio, television, newspaper or Internet/social media)
- health care system (e.g. campaigns or programs established in government policies, offered by clinicians or healthcare organizations such as counseling, informational/educational material, phone calls, multi-/inter-disciplinary teams, etc.)
- social/lay/peer (e.g. communication, strategies or programs offered by churches, community organizations such as cultural groups or immigrant settlement agencies, etc.)

**Immigrants**

- Immigrants are persons who come to live permanently in a foreign country
- Such studies will likely be based in Canada, United Kingdom, Europe (e.g. Nordic countries, Germany), Australia, New Zealand and United States, but might include other countries
- Refugees are a distinct group whose health issues may include trauma, infectious diseases, etc. so we will focus on immigrants. However, studies may use the label of immigrants or migrants, so we should search for both

**ELIGIBLE**

**Population**

- Adult immigrants aged 18+ years. Studies may focus on immigrants in general or women-only, both are eligible
- Immigrants as a group, or immigrants from specific countries or regions, or of a specific ethnicity/culture
- Immigrants who are well, or with specific disease/condition (e.g. diabetes, obesity, hypertension, breast cancer)
- Healthcare professionals (e.g. physicians of any specialty, nurses, personal support workers, physiotherapist, social worker, PA/exercise counselor, pharmacist, etc.) based in primary care, hospitals, community-based health centres/clinics
- Organizations (or individuals that work in/represent such organizations) that may function as conduits (i.e. knowledge brokers, change agents, champions, facilitators) or settings in which communication, social, lay or peer-led strategies or programs take place (might even include the work place)
- Government policy-makers or public health agency managers responsible for designing, implementing or evaluating PA health promotion programs
- If unsure whether population is comprised of immigrants, advance paper to full-text screening for further analysis

**Issue**

- Approaches, strategies, programs or interventions that promote PA or exercise to immigrants
- Strategies must be community-oriented, referring to delivery in community settings (including to the home) by healthcare professionals or community agencies or peer leaders
- Include studies with multifaceted interventions if it involves health promotion of PA as a factor
- Include studies that look at the enablers/ barriers of a specific health promotion tool or strategy of PA (not of the uptake of PA itself)
- Include studies that examine the development of a PA promotional tool or strategy
- SEE Concepts, Health Promotion for range of types of approaches, strategies, interventions
- SEE Concepts, Immigrants for PA health promotion targets

**Comparisons**

- Explore views about or optimal design of PA health promotion to immigrants among immigrants, healthcare professionals, government policy makers, public health managers or community organizations (or their representatives)
- Evaluate the use or impact of PA health promotion strategies/programs/interventions alone or in comparison with one or more other strategies/programs/interventions
- Assess determinants (enablers, barriers) of participation in, use or impact of strategies/programs/interventions

**Study Design**

- Empirical research – this means primary research that employed a specific research design and collected data (versus secondary analysis of published research, or articles that simply discuss a topic)
- Qualitative (interviews, focus groups, qualitative case studies, qualitative content analysis, descriptive investigations) or quantitative (questionnaires, before/after studies, prospective or retrospective cohort studies, case control studies, time series analysis, randomized controlled trials) or multiple/mixed methods studies
- Published in English language in any country
- Given that there may be few studies, and there is no particular date (that we are aware of at this time) that may have influenced PA among immigrants, we will search from inception of databases to current time
- Reviews (e.g. scoping, systematic) are not eligible but we will screen references for eligible primary studies

**Outcomes**

- Insight on desired or ideal design of PA health promotion to immigrants
- Enablers or barriers of participation in, use or impact of PA health promotion
- Effectiveness of PA health promotion to immigrants, assessed based on a range of possible impacts
- Impacts include, but are not limited to: knowledge/awareness/attitude/beliefs about the need for/importance of PA, help-seeking about PA (e.g. speaking to clinician or searching for information), intention to seek help/information, intention to undertake PA, actual practice of PA measured in a variety of ways (e.g. weight loss), uptake of PA, etc.

**NOT ELIGIBLE** (to be elaborated concurrent to screening titles/abstracts)

- Studies that focus on or largely include refugees (if studies are targeted to immigrants and refugees in general, we will include)
- Studies that focus on or largely include children or adolescents, as PA promotion strategies are likely to differ from those targeted to adults (e.g. school-based)
- Studies that describe enablers/barriers of PA (already established) versus PA health promotion (our focus)
- Studies that describe structured/facilitated PA or exercise programs versus PA health promotion (our focus)
- Syntheses (scoping, narrative, realist, systematic reviews) are not eligible, but we will screen the references of those on eligible topics
- Protocols, abstracts, editorials, letters, commentaries, or clinical guidelines
- Studies that focus on the education of specific diseases where promotion of PA is incidental
- Studies with poorly described methods and results that do not further examine the health promotion of PA
- Studies that focus on identifying the enablers/barriers to people engaging in PA
