# Supplementary material for: The design and impact of culturally-safe community-based physical activity promotion for immigrant women: descriptive review
Source: BMC Public Health. 2022 Mar 4;22:430. doi: 10.1186/s12889-022-12828-3 (PMC8895569; doi:10.1186/s12889-022-12828-3)
Supplement: Supplementary file 2 — Additional file 2. [file 12889_2022_12828_MOESM2_ESM.docx]

Additional File 2. MEDLINE search strategy

| **#** | **Search Statement** | **Results** |
| --- | --- | --- |
| 1 | "Emigrants and Immigrants"/ | 13065 |
| 2 | immigrant*.mp. | 31664 |
| 3 | newcomer*.mp. | 1579 |
| 4 | or/1-3 | 33035 |
| 5 | Exercise/ | 118673 |
| 6 | Physical Fitness/ | 28127 |
| 7 | physical activity.mp. | 121067 |
| 8 | exp Sports/ | 192379 |
| 9 | (physically adj3 active).mp. | 10206 |
| 10 | or/5-9 | 358360 |
| 11 | Health Promotion/ | 76419 |
| 12 | Health Education/ | 61748 |
| 13 | Patient Education as Topic/ | 86842 |
| 14 | Consumer Health Information/ | 4033 |
| 15 | Health Communication/ | 2713 |
| 16 | Counseling/ | 36993 |
| 17 | Public Health/ | 85625 |
| 18 | Preventive Health Services/ | 13916 |
| 19 | Primary Prevention/ | 19119 |
| 20 | Health Knowledge, Attitudes, Practice/ | 117406 |
| 21 | Health Policy/ | 68625 |
| 22 | Information Dissemination/ | 17794 |
| 23 | implementation science/ | 724 |
| 24 | "diffusion of innovation"/ | 17973 |
| 25 | Communication/ | 87706 |
| 26 | Information Seeking Behavior/ | 2737 |
| 27 | Social Networking/ | 4092 |
| 28 | Advertising/ | 14991 |
| 29 | exp Mass Media/ | 46365 |
| 30 | Communications Media/ | 1658 |
| 31 | exp Social Media/ | 10168 |
| 32 | Community Health Services/ | 32227 |
| 33 | or/11-32 | 684721 |
| 34 | 4 and 10 and 33 | 170 |
| 35 | limit 34 to (english language and "all adult (19 plus years)") | 125 |
| 36 | limit 35 to (comment or editorial or interview or lecture or letter or news or practice guideline) | 0 |
| 37 | 35 not 36 | 125 |
