# Supplementary material for: The design and impact of culturally-safe community-based physical activity promotion for immigrant women: descriptive review
Source: BMC Public Health. 2022 Mar 4;22:430. doi: 10.1186/s12889-022-12828-3 (PMC8895569; doi:10.1186/s12889-022-12828-3)
Supplement: Supplementary file 3 — Additional file 3. [file 12889_2022_12828_MOESM3_ESM.docx]

Additional File 3. Data extracted from included studies

| Study | Objective | Research design  Participants (n, % women) | Theory | Cultural safety | Results |
| --- | --- | --- | --- | --- | --- |
| Jih 2020 [31]  United States  Evaluate a PA promotion strategy  Community-based education (lay health worker) | Assess the impact of in-person community-based educational sessions + print material + follow-up phone call among Vietnamese Americans in Santa Clara County, California  ALSO: healthy eating | Randomized controlled trial (before/after questionnaire)  INT  2 educational sessions on physical activity  CON  2 educational sessions on colorectal cancer screening  n=627, 50.0% women | Diffusion of Innovations Theory | A physical activity flip chart was developed in Vietnamese using their vernacular, colloquial and idiomatic expressions and photos of active Vietnamese Americans by a team of bilingual and bicultural staff and consultants (and included in take-home booklet)  Immigrant settlement agencies recruited lay health workers who recruited participants and led sessions at the agencies in Vietnamese or English | Knowledge of physical activity recommendation increased from 2.6% to 62.4% among INT and from 1.0% to 2.5% among CON (p<0.001).  Self-reported physical activity of ≥ 150 min weekly increased from 28.9% to 54.0% in INT and from 38.0% to 46.8% in CON (p=0.001) |
| Weiland 2018 [32]  United States  Evaluate PA promotion strategy  Community-based education in the home (lay health worker) | Assess the impact of home-based in-person educational sessions + follow-up phone calls + print material for Hispanic, Somali, and Sudanese immigrants in Rochester, Minnesota  ALSO: healthy eating | Randomized controlled trial  (before/after questionnaire)  INT  6 educational sessions on physical activity + follow-up phone calls  CON  Delayed intervention as described above  n=70 (44 families), 60% women | Social Learning Theory | Bilingual family health promoters were trained to deliver the intervention to participants in their preferred language | Baseline physical activity values (based on accelerometer) were relatively high: Mean (SD) time spent per day in moderate to vigorous physical activity was 43.1 (35.4) minutes  No statistically significant differences were seen at 6 months (p=0.65) and 12 months (p=0.12) between the intervention group and controls for moderate to vigorous physical activity or sedentary time (p=0.81/p=0.19)  At 6 month/12 months, no significant differences were found in BMI (p=0.29/p=0.24), waist circumference (p=0.20/p=0.60), or blood pressure (p=0.11/p=0.13) in the intervention group compared with control group  For quality-of-life measures, non-significant trends toward improvement in physical well-being (p=0.25) and mood (p=0.27) were found at 12 months in the intervention group compared with controls and were sustained at 24 months |
| Jih 2016 [33]  United States  Evaluate a PA promotion strategy  Community-based education (lecturer + lay health worker) | Evaluate the impact of in-person, community-based educational sessions + print material + follow-up phone calls for older Chinese Americans in San Francisco, California  ALSO: healthy eating | Randomized controlled trial  (before/after questionnaire)  INT  2 educational sessions on physical activity + 2 follow-up phone calls + print material  CON  Print material only  n=725, 81% women | NR | A Chinese community–based organization helped to develop the lecture-based information and print materials. They reviewed lectures for cultural and linguistic appropriateness, culturally appropriate mnemonics to help participants remember the recommendations and culturally appropriate examples of physical activity  Chinese community-based organization recruited lay health workers who recruited participants and conducted follow-up phone calls. Sessions and content was in language of choice (Cantonese, Mandarin, or English). | The INT group had significantly greater increases (1.1% to 20.2%) than CON group (0.3% to 2.5%) in knowledge of physical activity (p<0.001).  Both INT (54.9% to 69.5%, p<0.001) and CON (54.9% to 64.7%, p=0.001) had significant increases in proportion of those reporting at least 150 min moderate physical activity weekly, but this was not significantly different between groups (p=0.20) |
| Ferrara 2015 [34]  United States  Develop a PA promotion strategy (assess views about use of social media)  Internet or Social Media | Explore facilitators and barriers of social media to promote physical activity to low English proficiency immigrants (largely Asian and Hispanic) in a New England city | Qualitative (focus groups)  Participants were asked about their knowledge and use of social media, physical activity, and how social networking could be used to promote physical activity  n=25, 68% women | NR | NR | - Knowledge and use of social media varied with some indicating frequent use of Facebook, Twitter, and Skype. For those, social media was used to connect with family and friends with whom they already had established relationships offline. - Perceived benefit of social media to share physical activity information: they could read information at their own pace to minimize the language barrier. - Perceived barriers that might limit their use of social media: access to technology or the internet, work and family obligations, privacy issues, and age. |
| Mitchell 2015 [35]  United States  Evaluate a PA promotion strategy  Community-based education (workplace, lay health worker) | Assess impact of community-based in-person educational sessions for Latino farmworkers at ranches in two California regions  ALSO: healthy eating | Randomized, controlled trial (before-after questionnaire and measurement)  INT  10 in-person group sessions on physical activity + commitment to change and review of compliance at each session  CON Participants received no intervention  n=178, 72% women | NR | A native of Mexico with expertise in adapting materials to lower literacy groups developed culturally and linguistically appropriate content  Lay health workers recruited participants and conducted educational sessions in Spanish language | Weight, BMI and waist circumference were significantly lower for INT compared with CON, particularly those who attended 8 to 10 of the 10 sessions (p=0.0002, p=0.0001, p=0.0012, respectively).  Number of days per week with at least 30 minutes of non-work physical activity days per week of moderate physical activity increased among INT group compared with CON (p=0.004, p=0.022, respectively)  Over 75% of INT group reported that they enjoyed making promises after each session  and reviewing them at the next session, and 87% thought the information presented was the most valuable aspect of the program |
| Samkange-Zeeb 2015 [36]  Germany  Develop a PA promotion strategy (assess views about design)  Internet or Social Media | Assess the acceptability of an educational “Internet health assistant” (web site to tailor guidance by sex, age or weight) for Turkish immigrants in one city in Germany  ALSO: healthy eating | Qualitative (one-day meeting + focus group)  Participants used the tool, discussed its acceptability and usability, and made suggestions for further improvement (n=8, 63% women) | NR | Turkish immigrants recruited through community organizations were consulted via interviews and home-based demonstration in three stages about typical sources of health information and preferred design of an Internet site for health information. Their feedback was used to refine the design. Content was bilingual | Participants identified the tool as being easy to use and navigate, and acceptable provided it were incorporated in existing technology such as smartphones. They welcomed the bilingual nature of the tool and found it useful that the tool also considered an individual’s context when giving information and/or advice. Participants indicated they would use the physical activity function in their everyday lives and that they preferred to get health advice from this tool more than from healthcare professionals. Participants suggested improvements to graphics, that the avatar trainer was not motivational enough, and to enhance cultural appropriateness (bilingual, reflect physical activity preferences) |
| Islam 2013 [37]  United States  Evaluate a PA promotion strategy  Community-based education (lay health worker) | Assess impact of educational sessions on Bangladeshi-Americans with Type 2 diabetes in New York City  ALSO: healthy eating | Before-after cohort study (survey, measurement, qualitative interviews with lay health workers)    10 monthly group sessions + 3 in-person home visits  n=26, 58% women | NR | Curriculum materials  were translated into Bengali by a certified translator and reviewed for accuracy by lay health workers and reps from community agencies  Curriculum integrated culturally relevant and tailored components such as gender specific exercises, and ways of negotiating social settings and situations particular to the community and study population  2 trained Bangladeshi lay health workers conducted sessions and home visits in Bengali language | Participants showed significant increases in knowledge of diabetes (p<0.001), physical activity as a way to control diabetes (p=0.05), and self-efficacy in health access (p<0.05). At 12-months, all participants were moderately or very confident that they could engage in physical activity regularly, and 89% of participants exercised at least several days a week compared to 52% at baseline. Changes in A1C, BMI, and weight were not significant.  A majority of individuals (ranging from 89% to 96%) reported that the number of educational sessions, session length, total months involved, and number of one-on-one visits were “just right” as opposed to “too many” or “too few.” All participants thought the program provided education, and social and peer support. Key barriers of participating in sessions included work conflicts (15%), transportation barriers (15%), and family obligations or influence (15%). Female participants often were not able to attend program components due to family obligations or lack of child care. Male participants, many of whom worked in service sector jobs such as taxi driving or restaurant work, were often restricted from attending sessions due to irregular and unpredictable work schedules.  Lay health workers were able to leverage unique access to and knowledge of community resources and shared participants’ cultural backgrounds, they were able to promote the study and increase acceptability. For example, they stated “people are willing to be more honest with me than they are with doctors” and “people feel confidence in me to share things.” They helped overcome language barriers for the largely limited English-proficient group and appealed to participants because they understood communal cultural norms. Home visits allowed lay health workers to view women in their home setting and provide tailored advice on how to make changes to physical activity, and also impact family by encouraging them to support their spouse. |
| Kandula 2015 [38]  United States  Evaluate a PA promotion strategy  Community-based education | Assess the impact of in-person educational sessions + behavioural counseling + phone follow-up for medically underserved South Asians (East Indian, Pakistani) at risk of cardiovascular disease in Chicago, IL  ALSO: healthy eating, stress management | Randomized control trial (before-after survey and measurement)  INT  6 weekly in-person group education sessions + follow-up phone support  CON  Translated print educational material  n=63, 63% women | NR | A community advisory board reviewed study materials and questionnaires  Sessions were delivered in a community organization in preferred language (Hindi or Urdu), and incorporated culturally relevant materials and experiences | INT group showed significant weight loss (-1.5 kg, p=0.04) and had a greater sex-adjusted decrease in hemoglobin A1C (-0.43 %, p <0.01) at 6 months compared with CON. There were no significant differences between groups for change in physical activity, blood pressure, total cholesterol, glucose levels or psychosocial outcomes (exercise-related confidence, coping) |
| Marcus 2015 [39]  United States  Evaluate a PA promotion strategy  Educational material (tailored to self-reported behaviour) | Assess the impact of mailed print educational material on physical activity for underactive Latinas (largely Dominican or Colombian) in Providence, RI | Randomized, controlled trial (before-after questionnaire)  INT  Regular mailings of physical activity brochures, tip sheets and individually tailored feedback reports  CON  Spanish-language pamphlets on heart-healthy behaviours other than physical activity (e.g. diet, smoking)  n=215 (100% women) | Social Cognitive Theory, Transtheoretical Model | Spanish-language material was culturally and linguistically adapted through the development phase based on input from the target population via 6 focus groups and 25 interviews to identify culture-specific attitudes and barriers to physical activity for Latinas: Participants preferred receiving physical activity information through mail-delivered print materials. Telephone contact was described as potentially disruptive to family time and many women expressed concerns regarding lack of Internet access. Anticipated barriers of physical activity were incorporated into the educational materials: balancing caregiver/household responsibilities, cultural norms about self-sacrifice, social support, partner negotiation, and dealing with inclement weather and neighborhood safety | INT group increased self-reported mean minutes/week of moderate to vigorous physical activity at 6 months (0.87 to 73.36 minutes per week, p<0.01) and 12 months (mean difference=51.99, SE 9.38, p<0.01) compared to CON group |
| Telle-Hjellset 2013 [40]  Norway  Evaluate a PA promotion strategy  Community-based education | Assess the impact of in-person educational sessions on Pakistani immigrant women in Oslo, Norway  ALSO: healthy eating | Randomized controlled trial (before-after measurement)  INT  6 in-person group educational sessions + 2 follow-up phone calls  CON  Single group session offering similar advice  n=198, 100% women | NR | Culturally-adapted pictures and figures were used because women had low levels of literacy. Session were conducted in community-based organizations in preferred language with multilingual personnel or with an interpreter. To facilitate physical activity, women were offered child care and good walking shoes. | There were no significant differences between CON and INT groups in any blood variables, blood pressure, BMI or waist circumference. Improvements from baseline to follow-up was significantly larger in the INT group compared with CON for fasting blood glucose and fasting insulin (between-group differences -0·16 mmol/l, p=0·003 and -5·8 pmol/l, P = 0·036). There were also small but significant reductions in glucose incremental AUC, 2h C-peptide and diastolic blood pressure, and fasting serum lipids in the INT group |
| Tovar 2012 [41]  United States  Develop a PA promotion strategy (assess views about design)  Community-based education | Develop an educational strategy to promote physical activity among new Latin and Haitian immigrants in the area of Somerville, Massachusetts  ALSO: healthy eating | Qualitative (planning meetings, focus groups)  Iterative co-design meetings with representatives of five community-based organizations, and focus groups with 5 Latina, 5 Brazilian, and 10 Haitian women shown a video of the first session of the curriculum  n=20, 100% women | Adult Learning Theory, Social Cognitive Theory | Design informed by prior focus groups with immigrant Spanish, Portuguese and Haitian-Creole women about barriers of physical activity and preferred types of interventions. They reported little control over time, little social support, and preferred group activities where they could interact with other women. Informed by focus group findings, researchers and leaders from five community-based organizations iteratively generated intervention design. Certified translators translated session content into Spanish, Portuguese, and Haitian Creole | 7 in-person group didactic and interactive educational sessions + 5 individualized follow-up phone calls using motivational interviewing. Sessions included discussion, finding solutions to problems, drawing, drama, physical activity, and other hands-on activities |
| Taylor 2008 [42]  United States  Develop a PA promotion strategy  Community-based education | Develop an educational session on physical activity for Chinese immigrants | Qualitative (focus groups)  Four focus groups: 2 in Seattle with 12 Cantonese-speaking men and 11 Mandarin-speaking women, and 2 in Vancouver with 12 Cantonese-speaking women and 11 Mandarin-speaking men  n=46, 50% women | NR | NR | Desired information:   - Benefits of physical activity - Negative consequences of inactivity - Recognize traditional Chinese beliefs about the appropriateness of vigorous physical activity for older people - Address common barriers (lack of time, work schedules, weather conditions, safety concerns, lack of familiarity with location of amenities, cost of equipment) - Advise on relevant community resources   Desired design:   - Interactive class exercises - Audio-visual presentation - Use of visual aids - Content includes advice on warm-up exercises, vocabulary cards, information-gap worksheets, videos, guided discussion exercises and problem/advice cards |
| Kim 2004 [43]  United States  Evaluate a PA promotion strategy  Community-based education (lay health worker) | Assess the impact of in-person education sessions on low-income Latin immigrants in Los Angeles, California  ALSO: healthy nutrition, smoke-free environment | Before-after cohort study (questionnaire, qualitative interviews)  3 in-person educational sessions in Spanish language on physical activity  n=256, 98% women (interviews with 33 women) | NR | Intervention design was informed by an advisory board with representatives of two community-based organizations. They recommended use of promotoras, reviewed program content, and advised on recruiting lay health workers. Lay health workers (n=9) were bilingual who had an intimate understanding of community sociocultural background, experiences and challenges. They were trained to recruit participants and teach sessions, which were delivered in Spanish language and conducted in school-based parent centers, community centers, church, workplace, and lay health worker home. | Overall lifestyle behaviours (t= –13.40, p<0.011) and physical activity behavior (t= –12.46, p<0.001) improved significantly. For physical activity, participants reported a sense of well-being, weight loss, successfully incorporated family members into an exercise routine, and integrated a daily routine into an exercise routine. Challenges they faced included managing family resistance to exercise and time limitations |
