# Supplementary material for: The design and impact of culturally-safe community-based physical activity promotion for immigrant women: descriptive review
Source: BMC Public Health. 2022 Mar 4;22:430. doi: 10.1186/s12889-022-12828-3 (PMC8895569; doi:10.1186/s12889-022-12828-3)
Supplement: Supplementary file 4 — Additional file 4. [file 12889_2022_12828_MOESM4_ESM.docx]

Additional File 4. PA promotion strategy design

| Study | Content | Format | Delivery | Timing | Personnel |
| --- | --- | --- | --- | --- | --- |
| Jih 2020 [31]  United States  Community-based education (lay health worker) | INT  Session #1: Information on physical activities commonly done by Vietnamese Americans supported by bilingual flip chart with images of Vietnamese Americans participating in physical activity. Session #2: Lay health workers identified participants who had not met recommended physical activity behaviors, identified barriers to change and provided suggestions and support to overcome these barriers. Sessions and content was bilingual (Vietnamese, English).  CON  2 sessions similar to above focused on colorectal cancer screening | Didactic and interactive educational sessions based in immigrant settlement agencies | In-person group session + same information in take-home booklet + follow-up phone reminders (or in-person visits) | Sessions were 1 to 2 hours in length. Session #2 took place 2 to 3 months after the first. | Vietnamese American lay health workers were non-healthcare professionals recruited through settlement agencies. They were trained to:   - Recruit participants from among family, friends and referrals - Deliver educational sessions - Distribute booklets - Conduct follow-up phone calls |
| Weiland 2018 [32]  United States  Community-based education (home, lay health worker) | INT  4 physical activity sessions addressed: increasing physical activity, muscle strength and flexibility, reducing screen time, and overcoming barriers to physical activity + 2 reinforcing sessions on exercise/work-life balance and celebrating accomplishments.  CON  Delayed intervention as described above | Didactic and interactive sessions based in home | In-person individualized sessions in participants’ preferred language (Cantonese, Mandarin, or English) + follow-up phone calls + print material (list of community resources for physical activity; newsletter of key messages and success stories) | 4 sessions + 2 summary sessions of 60-90 min over 6-month period + up to 12 15-minute follow-up phone calls every 2 weeks within a second 6-month period | Trained bilingual family health promoters of Hispanic, Somali, and Sudanese origin delivered sessions and used role-modeling, feedback and reinforcement, shaping, and social support to enhance self-efficacy and behavior change |
| Jih 2016 [33]  United States  Community-based education (lecturer + lay health worker) | INT  Lecture #1: focused on physical activity education that was tailored to older Chinese Americans and used culturally appropriate examples of physical activity. Lecture #2: general information on hypertension, hypercholesterolemia, and diabetes mellitus; how they affected Chinese Americans; and how to reduce the risks of those conditions. Print material offered same content as lectures. Sessions/content was in language of choice (Cantonese, Mandarin, or English).  CON  Same print material as above. They also received 2 lectures and 2 follow-up phone calls from lay health workers about colorectal cancer screening | Didactic educational sessions in Chinese community-based agency | In-person group sessions + same information in take-home print material + follow-up phone calls | Two 60–90-minute lectures delivered about 2 months apart; 2 follow-up phone calls one month following each educational session | Lecturer conducted educational sessions. Lay health workers conducted follow-up phone calls |
| Mitchell 2015 [35]  United States  Community-based education (workplace, lay health worker) | INT  Session were conducted in Spanish and topics included a program overview, increasing physical activity, what is a healthy weight, understanding and preventing diabetes, and the connection between feeling good, being healthy, mental health, diet and exercise. At each session, participants were asked to promise to implement learning and improve lifestyle. At the beginning of each session, if someone had trouble keeping their promise, the group would jointly devise solutions  CON  No information | Didactic and interactive educational sessions | In-person group sessions + commitment to change and review of compliance at each session | 10 sessions (9 educational 1 final review, each 90 min in length including a 15-20 minute physical activity (warm-up, moderate activity, cool-down) over a 12-week period of time | Trained lay health workers (promotoras) recruited participants and conducted the sessions |
| Islam 2013 [37]  United States  Community-based education (lay health worker) | Sessions were conducted in Bengali and for men- and women-only. Sessions included information, group activities and physical exercise. The first session provided an overview of diabetes, including myths and facts, disease specific information, and blood glucose levels. The following sessions included topics on physical activity, diabetes, stress and family support, and access to health care. The one-on-one sessions were focused on participant challenges and strategies for diabetes management | Didactic and interactive educational sessions | In-person group sessions + in-person individualized home visits | 6 monthly, 2.5-hour group sessions and 3 one-on-one visits from lay health worker in months 3, 6, and 9 of 60-90 minutes in length | Trained lay health workers (one male, one female) conducted sessions and home visits |
| Kandula 2015 [38]  United States  Community-based education | INT  6 weekly educational sessions in preferred language (Hindi or Urdu) on #1: What is Heart Disease and Understanding Your Risk Factors; #2: How to Get More Exercise; #3: Eat Less Fat and Salt; #4: Enjoy Fruits, Vegetables, & Grains; #5: Maintain a Healthy Weight; #6: Taking Care of Stress and Tension). Were taught about national physical activity guidelines. They were given pedometers, and were taught how to self-monitor daily steps and how to gradually increase activity. Participants were encouraged to set realistic behavior change goals based on their current behaviors.  CON  Monthly mailing of the National Heart, Lung, and Blood Institute's print education materials on heart disease, diet, exercise, and weight (translated into Hindi and Urdu) | Didactic and interactive sessions: participants watched the video pertaining to the class topic, followed by discussion, experiential activities, goal-setting, and closing review | In-person group sessions + follow-up phone calls based on motivational interviewing to focus on self-reflection, behavior goals, and problem solving | Weekly session for 6 weeks of 60-90 minutes. Phone calls started after classes ended and continued for 10 weeks. | NR (likely study personnel) |
| Marcus 2015 [39]  United States  Mailed print educational material | INT  Spanish-language motivation-matched physical activity manuals, tip sheets and individually tailored feedback reports generated by a computer expert system based on based on participants’ responses to monthly questionnaires that draws from 296 messages regarding motivation, self-efficacy, and cognitive and behavioral strategies for physical activity adoption. The expert system also provides feedback on progress over time and compared to other individuals who successfully adopted and maintained physical activity. Participants were also given information on local physical activity resources, pedometers, and 12 physical activity logs.  CON  Spanish-language pamphlets on heart-healthy behaviours other than physical activity (e.g. diet, smoking) | Educational print materials in Spanish on physical activity guidance + tailored feedback | Mailed print educational material | 11 mailings in first 6 months, then tapered over next 6 months (4 mailings in month1, 2 mailings in months 2 and 3, 1 mailing in months 4 to 6) with booster mailings in months 8, 10 and 12 | Study personnel developed material, informed by prior research involving interviews with Latinas |
| Telle-Hjellset 2012 [40]  Norway  Community-based education | INT  Sessions were offered in preferred language. The main focus was on the physiological importance of blood glucose and its regulation by diet and physical activity, and on knowledge about the Pakistani lifestyle. Session topics included the beneficial effect of light physical activity, especially post-meal walking. Women were also encouraged to walk for 1 hour twice per week  CON  Single group session offering similar advice | Didactic and interactive educational sessions | In-person group sessions + single brief individual feedback after first session on diet and physical activity + scheduled walking groups + child care + good walking shoes | 6 sessions of 2 hours involving 10 to 12 women each over 7 months + 2 follow-up phone calls 1 months after each session | Female research personnel delivered the intervention with the aid of multilingual personnel and interpreters |
| Kim 2004 [43]  United States  Community-based education (lay health worker) | Session #1 - Lay health workers explained the study and participants completed a baseline questionnaire  Session #2 and #3 - Lectures and materials on physical activity were provided. Content included a video on physical activity and audio-taped activity instructions with music and picture cards. Sessions were delivered in Spanish language | Didactic educational sessions | In-person group sessions | 3 2-hour sessions offered weekly | Trained lay health workers were peer members of the community with lived experience of being Latinx in an underserved community. They were trained to recruit participants from among their social networks and conduct educational sessions |
